# Exploring the limits of conventional small-scale CHO fed-batch for accelerated on demand monoclonal antibody production

**DOI:** 10.1007/s00449-021-02657-w

**Published:** 2021-11-09

**Authors:** Amélie Mahé, Alexandra Martiné, Séverine Fagète, Pierre-Alain Girod

**Affiliations:** Selexis SA, 36 route de la Galaise, CH-1228 Plan les Ouates, Switzerland

**Keywords:** CHO cell culture, Intensified fed-batch, ambr15, Productivity, Biomass

## Abstract

In the field of therapeutic antibody production, diversification of fed-batch strategies is flourishing in response to the market demand. All manufacturing approaches tend to follow the generally accepted dogma of increasing titer since it directly increases manufacturing output. While titer is influenced by the biomass (expressed as IVCD), the culture time and the cell-specific productivity (*q*_*P*_), we changed independently each of these parameters to tune our process strategy towards adapted solutions to individual manufacturing needs. To do so, we worked separately on the increase of the IVCD as high seeding fed-batch capacity. Yet, as intensified fed-batch may not always be possible due to limited facility operational mode, we also separately increased the *q*_*P*_ with the addition of specific media additives. Both strategies improved titer by 100% in 14 days relative to the standard fed-batch process with moderate and acceptable changes in product quality attributes. Since intensified fed-batch could rival the cell-specific productivity of a conventional fed-batch, we developed novel hybrid strategies to either allow for acceptable seeding densities without compromising productivity, or alternatively, to push the productivity the furthest in order to reduce timelines.

**Supplementary Information:**

The online version contains supplementary material available at 10.1007/s00449-021-02657-w.

## Introduction

With the advent of unnatural molecules that pose increasing industrial problems as these have unpredictable manufacturability, the protein therapeutics industry must constantly innovate. Many bioprocess optimization strategies have been explored to increase the volumetric productivity while maintaining low operational complexity. Monoclonal antibodies (mAbs) have benefited most from these continuous efforts to meet increasing market demand and reduce manufacturing costs [1, 2].

The preferential mammalian system of expression of biopharmaceuticals are the Chinese Hamster Ovary cells (CHO) for their ability to perform post-translational modifications close to human, its efficient protein folding and its delivery of high productivity [3–5]. Over the last 2 decades, expression of mAbs in CHO has been greatly improved and volumetric titers of 10 g/L in 14–18 days can now be achieved [6–9]. These titers are associated with the most dominant production method for mAbs, the fed-batch mode [8–11].

Fed-batch mode is a simple production mode in which a basal medium is supplemented with feed solutions containing nutrients to support the production phase. It is well recognized for its high mAb yields, its reliability, ease of execution and implementation at the industrial scale [9]. In addition, multiple parameters such as basal and feed media components, feeding strategy, temperature, pH or process duration can be optimized to maximize cell culture longevity and increase productivity. Nevertheless, due to the increasing demand for improved efficiency, alternative fed-batch modes have emerged. Most of them are qualified as fed-batch process intensification strategies due to the achievement of high seeding densities. A recent concept consists in pre-stage perfusion (N–1) which allows for robust accumulation of biomass by cell retention for higher seeding density of bioreactors (N) [12–14]. It results in shorten production timelines by reducing process duration or can lead to higher final harvest titer in smaller bioreactors. Interestingly, this hybrid operational mode moderately impacts facility design, as it is based on pre-existing processes rather than implementing completely new technologies. Saving time, space and money are additional gains to enhanced productivity.

Despite these great advances in manufacturing, there are still process improvement at early stage, notably, for early production. Cell line providers are at the interface of drug development. Not only they provide stable expressing cell lines by selecting highly productive clones, but they must also ensure early process transfer. If flexibility would be introduced at this stage, it would help to better satisfy the future needs of the production of the drug. Different considerations must be taken into account. First, technical restrictions such as support technologies available at the contract manufacturing organization (CMO) or facility design will help to pick the best operational mode [15, 16]. Second, requirement for accelerated timelines to achieve a faster delivery of the pipeline to circumvent competitors or in response to urgent pandemic outbreak needs might also dictate the appropriate process [17]. Third, depending on the clinical application either product quantity or quality may have an impact on the choice of the process mode or duration [18, 19]. In a word, anticipating these different demands at the earliest is turning critical and adapted early process solutions are expected.

In this study, we aimed at bringing this open mind set flexibility in cell line development to be able to adapt early fed-batch process solutions. Both the biomass (expressed as the integral of viable cell density, IVCD) and the specific productivity of the cells (*q*_*P*_) are positively correlated with final product titer.

(Titer = *q*_*P*_ × IVCD) [20]. We successfully influenced the expression titer of two model mAbs (mAb A and mAb B) above 10 g/L in 14 days, solely by acting on either an increased biomass reaching 50 × 10^6^ cells/mL peak density or by ameliorating *q*_*P*_ (40 pg.cell-1.day-1) using a chemical engineering approach. Furthermore, we demonstrated that *q*_*P*_ was not improved at the expense of product quality as only moderate changes in product quality attributes were observed. Based on these findings, we developed novel hybrid strategies, closest to manufacturing demand, to either allow for acceptable seeding densities without compromising productivity, or alternatively, to push the productivity the furthest to shorten the production timelines. Our study demonstrates the undeniable plasticity of the platform’s process parameters and highlights that comprehension of consecutive stages of mAb development, is key determinant to provide new adapted and on-demand CHO fed-batch early process solutions.

## Materials and methods

### CHO-M cell line and batch cultivation

Distinct Selexis CHO-M producing cell lines stably expressing two different human monoclonal IgG1 antibodies, respectively, named mAb A and mAb B were initially chosen for intensified fed-batch development. An enlarged panel of cell lines producing recombinant proteins of different nature (mAbs, Fc-fusions, bispecifics, and others) were also tested in the different fed-batch processes to assess systematic process applicability and determine the impact on productivity. At the start of each process campaign, one cell vial was thawed, seed train cultures were passaged every 3–4 days prior to N-1 seed. Four days before microbioreactor inoculation, CHO-M producing cell line cultures were passaged in shake flasks at a seeding cell density of 0.30 × 10^6^ cells/mL (N-1) at a volume according to process needs (Supplementary Fig. 1). Cells were cultivated in the chemically defined BalanCD Growth A culture medium (FUJIFILM Irvine Scientific, USA) supplemented with 6 mM L-Glutamine (HyClone, USA) with incubator (Kühner, Germany) settings at 37.0 °C, 5% CO_2_ and 120 rpm.

### Fed-batch cultures and in-process cell culture analytical methods

Cell growth and production performance were evaluated using the ambr15 automated microscale bioreactor system (Sartorius Stedim, Germany) equipped with a cooling system to allow temperature shift. All cultures were carried out with 40% of dissolved oxygen (DO), stirring speed between 1000 and 1400 rpm, temperature maintained at 36.5 °C then shifted at 33.0 °C (time shift according to seeding density) and pH controlled at 6.90 ± 0.10 then shifted at 7.00 ± 0.20 using CO_2_ and 1 M carbonate (time shift according to seeding density). Microbioreactors were seeded at a target cell density of 0.45, 1.00 or 10.00 × 10^6^ cells/mL in different initial working volume depending on the seeding density process. Cell Boost 7a and Cell Boost 7b feed supplements (HyClone, USA) were added to cultures at various days, depending on the seeding density process. Glucose solution (Sigma Aldrich, USA) was added based on daily glucose concentration to bring glucose level back to 6 g/L and when trigger limit of 4 g/L was reached. Copper acetate (Sigma Aldrich, USA) supplement was added during the early cell growth phase [21–24]. Ferric citrate (Sigma Aldrich, USA) supplement was added during the late cell production phase [21, 25–27]. Initial working volume and process additives volumes (commercial feeds and metal supplements) were adjusted all along the process duration according to biomass and culture phase. Volume distributions (feeding versus sampling) comparisons showed that the total feed addition balances the samples collection and that no difference in volume is observed within the different processes (Supplementary Fig. 2a). In addition, usual working volume ranges specified by the supplier have been respected (Supplementary Fig. 2b). Microbioreactor samples were harvested daily for immediate cell counting, metabolites, pH and gas analysis. VCD, viability, glucose, lactate, glutamine, glutamate, ammonia, pH, pO_2_, and pCO_2_ were measured using a Bioprofile FLEX2 (Nova Biomedical, USA). Cells were grown for 14 days.

### Off-process product characterization assays

Microbioreactors were sampled daily for antibody quantification by Protein A-high-performance liquid chromatography (HPLC) (ThermoFisher, USA). Harvest was collected either at day 7, 10 or 14 and clarified for product quality characterization. Antibody purification was performed using centricolumns (Repligen, USA) filled with Protein A resin. Purified IgG was buffer-exchanged with PBS for quality attributes analysis including level of size variants, charge heterogeneity and N-linked glycosylation. High molecular weight (HMW) species were measured using High Performance Size Exclusion Chromatography (SEC-HPLC) (ThermoFisher, USA). Low molecular weight (LMW) species were determined using Micro-Capillary Electrophoresis (µCE) with LabChip® GXII Touch™ instrument (PerkinElmer, USA) [28]. Charge variant (main peak level, acidic and basic isoforms) analysis was performed using imaged capillary isoelectric focusing (icIEF) technology with Maurice instrument (Protein Simple, USA). N-Glycans were released from Protein A purified antibody using PNGase F (Promega, USA). Released oligosaccharides chains were labeled with APTS (Sigma Aldrich, USA), separated on a Capillary Electrophoresis (CE) (SCIEX, USA) and detected by Laser-Induced Fluorescence (LIF) at 488 nm. Glycans were assigned according to commercial libraries (e.g. Man5, G0, G0F, G0F^−^N, G1Fa, G1Fb and G2F) (Prozyme, USA).

## Results

### Increasing the biomass

**Fig. 1:**
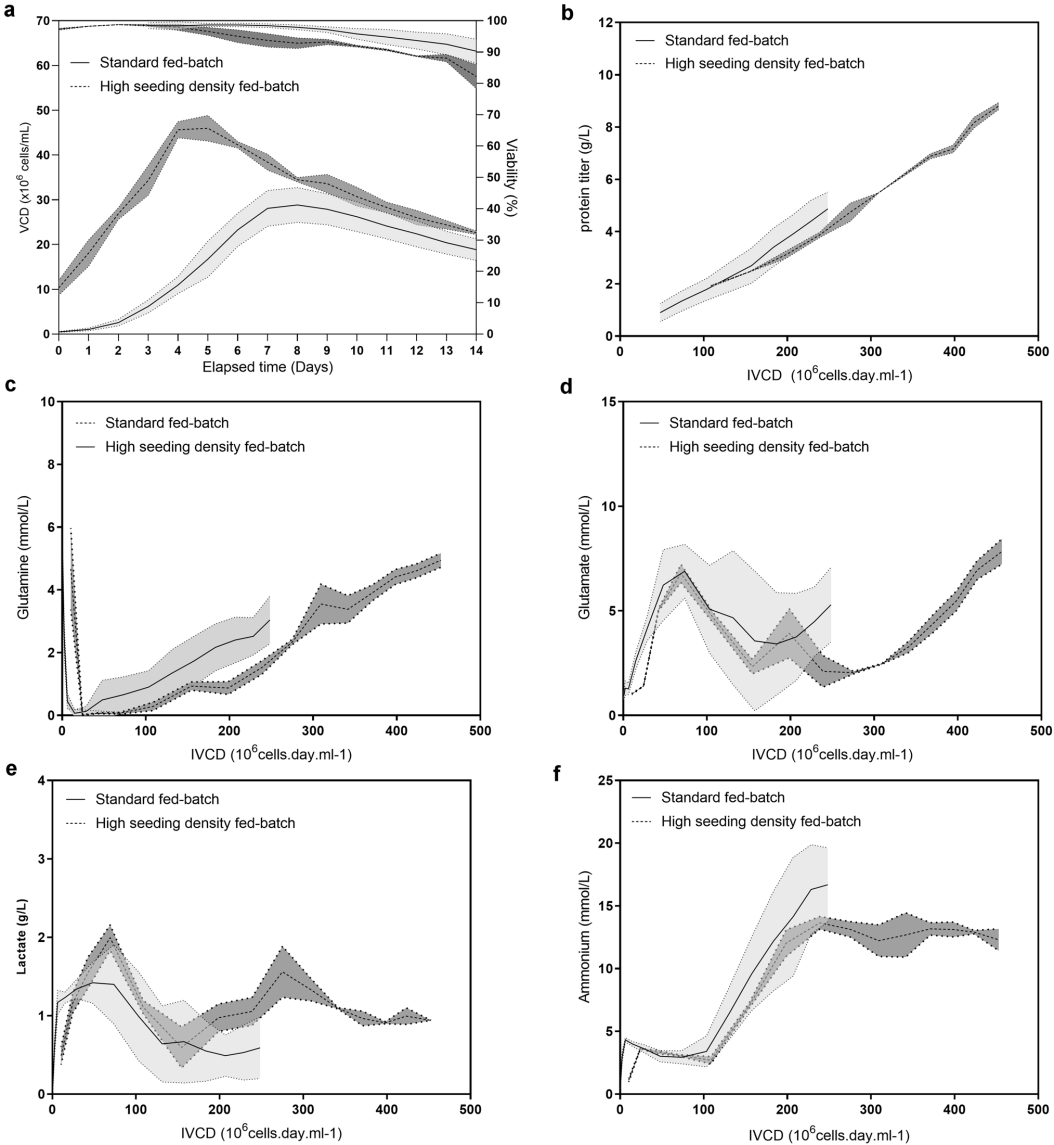
Fed-batch process performance playing with biomass. VCD and viability (**a**), specific productivity (**b**), glutamine (**c**), glutamate (**d**), lactate (**e**), and ammonium (**f**) profiles of standard fed-batch expressing mAb A inoculated with starting seeding density of 0.45 ± 0.1 × 10^6^ cells/mL (*n *= 14, black solid line with SD represented with light grey errors bands) and high seeding density fed-batch inoculated with starting seeding density of 10 ± 1.7 × 10^6^ cells/mL (*n *= 2, black dashed line with SD represented with dark grey errors bands)

In this study, we aimed at exploring different paths to influence expression titer during a fed-batch. Our first assumption was the capacity to enhance expression titer by increasing the total number of producing cells. We, therefore, examined the effects of increasing inoculation densities to reach a higher biomass and potentially improve the final harvest titer. Using mAb A as a model, the initial seeding density of the standard fed-batch was intensified from 0.45 ± 0.1 × 10^6^ cells/mL (*n *= 14) to 10 ± 1.6 × 10^6^ cells/mL (*n *= 2) for high seeding fed-batch (Fig. 1a). The peak VCD for standard fed-batch was 29.6 ± 3.8 × 10^6^ cells/mL on day 8 or 9 and the final VCD on day 14 was 18.9 ± 2.4 × 10^6^ cells/mL. High seeding fed-batch reached a higher peak VCD of 46.2 ± 2.6 × 10^6^ cells/mL on day 5, and a final VCD on day 14 of 22.6 ± 0.4 × 10^6^ cells/mL. As the setpoint for temperature time shift was defined according to the cumulated biomass, the peak VCD was not reached at the same time for both seeding densities. Indeed, the aim behind defining the temperature shift set point was to maintain cells in a production state. Therefore, temperature shift occurred on day 5 for standard fed-batch, while on day 3 for high seeding density fed-batch. In both processes, the peak VCD was reached 2 days post-temperature shift. Cell viability was well maintained for standard fed-batch (90.3 ± 3.8% on day 14), while the cell viability for high seeding density fed-batch was slightly lower (82.2 ± 3.8% on day 14). However, the lower cell viability of the high seeding density fed-batch did not have any negative impact on process performance. Consequently, increased initial cell density led to the intensification of productive biomass expressed as integral viable cell density (IVCD) and calculated as follows:

1 $$IVCD = \frac{{VCD_{Dx} - VCD_{Dx - 1} }}{{\ln \frac{{VCD_{Dx} }}{{VCD_{Dx - 1} }}}} \times \left( {T_{Dx} - T_{Dx - 1} } \right) + IVCD_{Dx - 1} ,$$

where *VCD*_*Dx-1 or Dx*_ (10^6^ cells.days.mL^−1^) are the viable cell densities of the last and the current monitored points, respectively, *T*_*Dx-1 or Dx*_ (day) are the time points for the last and the current monitored points, respectively, and *IVCD*_*Dx-1*_ (10^6^ cells.days.mL^−1^) is the integral viable cell density for the last monitored point.

Meanwhile, we redesigned the feeding regimen to maintain an optimal biomass productivity. In high seeding fed-batch, the feeding regimen was initiated from day 1 and was then daily adjusted for the rest of the fermentation time according to both the biomass (VCD) and the culture growth phase (lag phase, exponential phase, stationary phase, death phase), whereas in standard fed-batch, nutrient addition started on day 3. The feed strategy for both processes focused on delivery of sufficient amount of nutrients to keep a balance between cell growth and protein production. Consequently, we observed a continuous linear accumulation of product expressed by the cell in both processes (Fig. 1b). As a result, a final titer of 8.8 ± 0.1 g/L of mAb A was reached on day 14 in the high seeding fed-batch, which was 1.8 higher than the standard fed-batch titer of 4.9 ± 0.6 g/L on day 14. For both conventional fed-batch process and intensified fed-batch process, microscale bioreactors displayed similar profiles for key nutrient residual concentration (i.e. glutamine and glutamate) and the concentration of some metabolic by-products (i.e. lactate and ammonium) relative to the cell biomass (Fig. 1c, d, e, f).

This first set of experiment demonstrated that by increasing the biomass and adjusting the feed regimen solely, we were able to sustain the productivity and hence increase the final titer, while leaving other monitored metabolites profiles unchanged. We demonstrated that process parameters have been successfully adapted to high seeding density small bioreactor fed-batch without affecting the metabolic flux performance leading to a continuous accumulation of the product.

### Increasing ***q***_***P***_

After demonstrating that high-density fed-batch process achieved high titer keeping very good cell growth during the 14 days production time, we next sought to evaluate if the cell-specific productivity (*q*_*P*_ calculated as detailed below) could achieve even better performance.

2 $$q_{P} = slope\frac{{\Delta \left[ {Titer} \right]}}{\Delta IVCD} \times 1000.$$

**Fig. 2:**
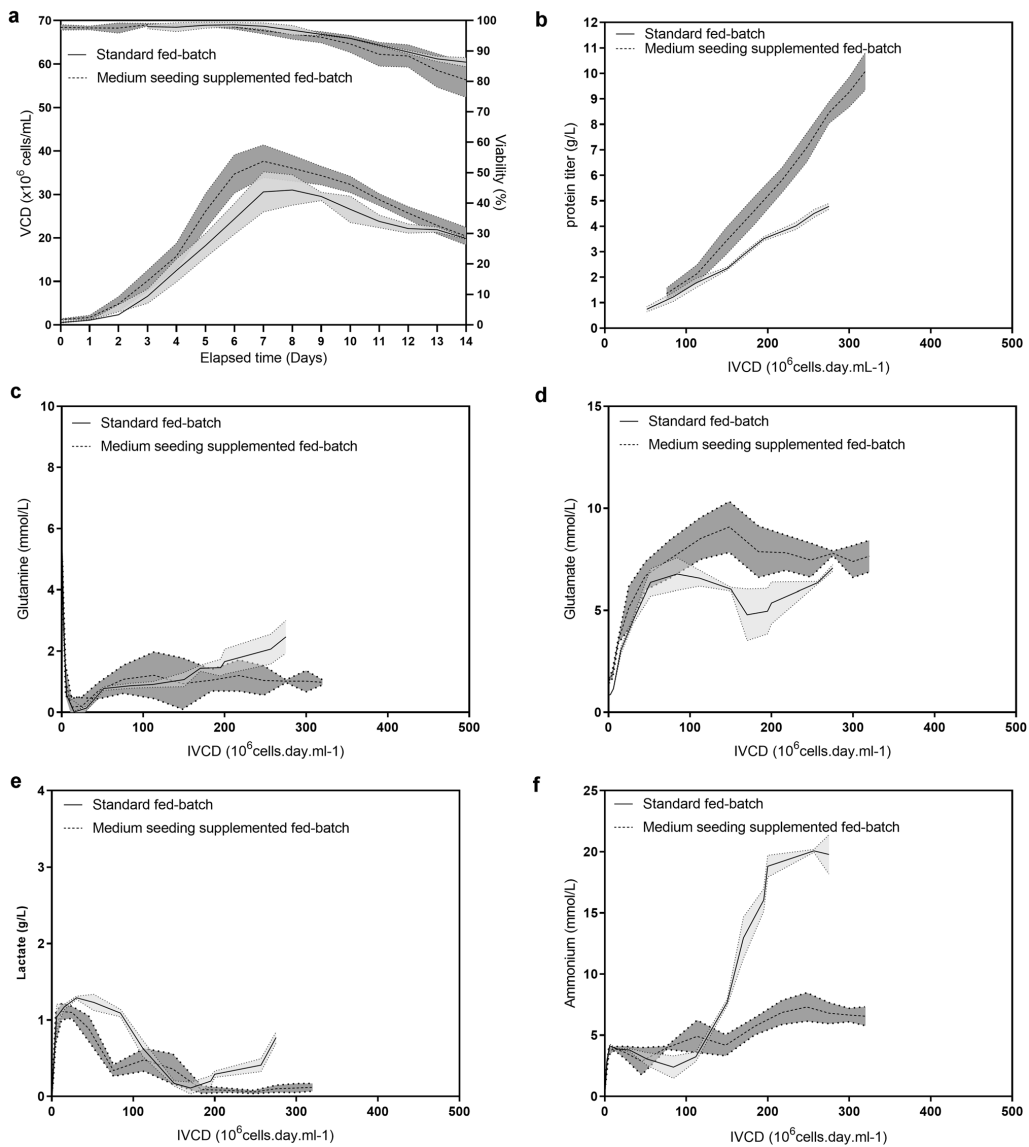
Fed-batch process performance playing with *q*_*P*_. VCD and viability (**a**), specific productivity (**b**), glutamine (**c**), glutamate (**d**), lactate (**e**), and ammonium (**f**) profiles of standard fed-batch expressing mAb B inoculated with starting seeding density of 0.45 ± 0.1 × 10^6^ cells/mL non supplemented (*n *= 3, black solid line with SD represented with light grey errors bands) and medium seeding density fed-batch inoculated with starting seeding density of 1 ± 0.2 × 10^6^ cells/mL and supplemented with copper acetate and ferric citrate (*n *= 5, black dashed line with SD represented with dark grey errors bands)

Indeed, at commercial stage high-density fed-batch production modes require a large biomass for inoculation. This required cell mass remains easily handled at small scale but become a significant limitation when large culture volume is needed. Therefore, by taking into consideration the technical constraint of high seeding fed-batch, we explored a minor increase of initial seeding density combined with supplementation of cell culture production media to increase final harvest titer. In this second set of experiment, the initial seeding density was increased from 0.45 ± 0.1 × 10^6^ cells/mL to 1 ± 0.2 × 10^6^ cells/mL (Fig. 2a) for production of mAb B. The classical feed strategy (Cell Boost 7a and Cell Boost 7b feeds) was adapted to sustain both cell growth and cell productivity as described previously. Additionally, two supplements, copper acetate and ferric citrate, were added to boost the *q*_*P*_ in medium seeding supplemented fed-batch [23, 26]. Copper acetate was added by bolus addition during the early phase, whereas ferric citrate was added in the late production phase [21–23, 26, 27]. The medium seeding supplemented fed-batch reached a higher peak VCD of 37.6 ± 3.7 × 10^6^ cells/mL as compared to the 31.2 ± 3.6 × 10^6^ cells/mL reached in the standard fed-batch. Thus, the medium seeding fed-batch had a higher final VCD on day 14 of 20.4 ± 2.1 × 10^6^ cells/mL compared to 19.9 ± 0.2 × 10^6^ cells/mL of the standard fed-batch. Viability also showed good profile for both fed-batch ending, respectively, at 86.3 ± 1.5% and 80.5 ± 5.7% for standard and medium seeding density culture. We found that the metals combination had a significant effect on cell culture performance (e.g. peak VCD, titer). The titer increase was mainly due to an increase in the cell-specific productivity (Fig. 2b). The rise of linear slope from the plot of product concentration versus IVCD (from 20.3 ± 3.3 to 36.9 ± 1.8 pg.cell-1.day-1) resulted in a 2.2-fold increase in the final harvest titer of standard fed-batch (4.7 ± 0.1 g/L) compared to the supplemented medium seeding fed-batch (10.1 ± 0.7 g/L). Glutamine, glutamate, and lactate profiles were comparable overtime between the two processes, while the toxic metabolite ammonium decreased (Fig. 2f) reflecting an adapted energy metabolism that benefited the cell culture performance.

In summary, we found that supplementation of the production media combined with medium inoculation density had an additive effect on improving titer and cell-specific productivity. Using conventional fed-batch process, we demonstrated that large-scale acceptable seeding density of 1.00 × 10^6^ cells/mL supplemented with optimized additives can exceed final titers of 10 g/L for mAb B.

### Towards an on-demand, tuned production process

**Fig. 3:**
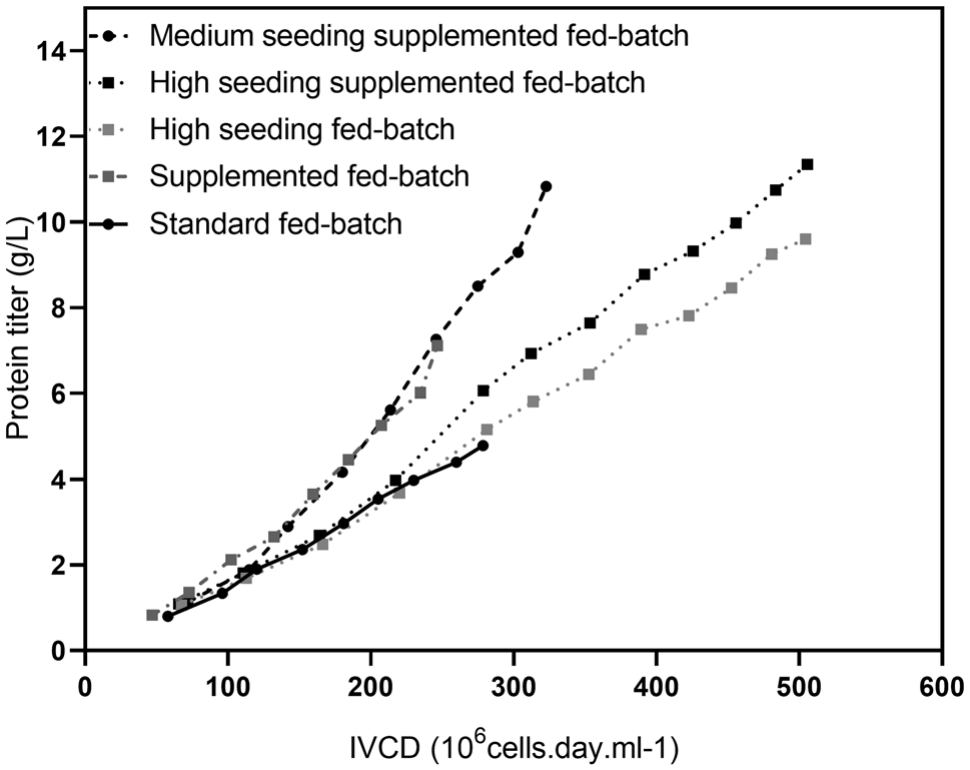
Productivity performance of different intensified processes. mAb B *q*_*P*_ of the four intensified processes (dashed lines) compared to the initial standard fed-batch (black solid line)

**Fig. 4:**
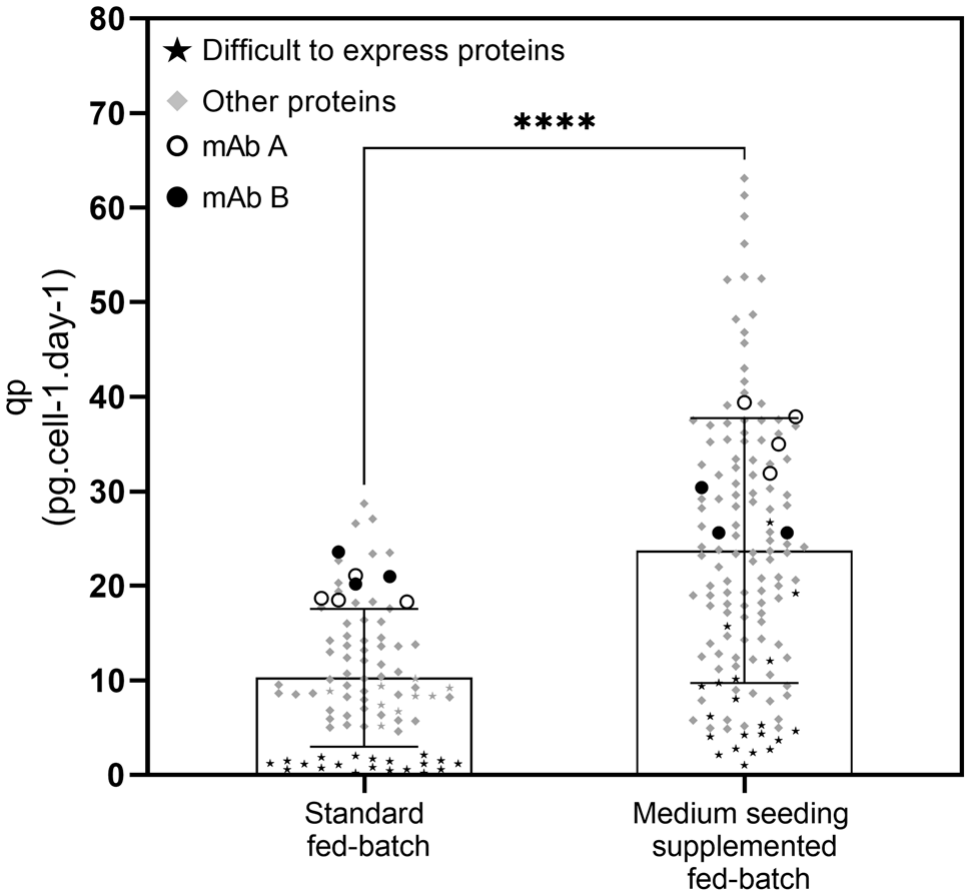
Intensified process impact on specific productivity of different CHO-M producing cell lines. Specific productivity data of standard and medium seeding supplemented fed-batch from enlarged panel of CHO-M producing cell lines expressing proteins of different nature: mAbs, Fc-fusions, bispecifics and difficult to express proteins (*q*_*p*_ < 2 pg.cell-1.day-1). The *p* value is less than 0.0001 and by conventional criteria, the difference in *q*_*p*_ between processes is considered extremely statistically significant

Taking into consideration the data provided above, both strategies, either high-density inoculation or supplementation, appear to be technically adapted to small-scale fed-batch of early stages of mAb development. Indeed, the use of these two levers for improvement create flexibility in designing early fed-batch process solutions. Figure 3a summarizes the productivity performance of all intensified processes expressing mAb B playing on IVCD, *q*_*P*_ or simultaneously on both parameters. Depending on the manufacturing constraint, a conventional initial seeding (0.45 × 10^6^ cells/mL) can be a requirement thereby, a *q*_*P*_ increase from 18.3 to 30.2 pg.cell-1.day-1 on standard fed-batch with supplementation allow 1.5-fold titer increase on day 14. Additionally, with high seeding fed-batch, the specific productivity is maintained over production time (respectively, 18.3 and 20.5 pg.cell-1.day-1 for standard and high seeding fed-batch) enabling 2.0-fold increase in the final harvest titer. Combining both strategies, the specific productivity for supplemented high seeding fed-batch was increased from 18.3 to 24.3 pg.cell-1.day-1 reflecting a final titer improvement from 4.8 to 11.4 g/L, i.e. a noteworthy 2.4-fold titer increase. Nevertheless, the combination of supplementation and seeding increase is even more pronounced on intermediate seeding inoculation: *q*_*P*_ increases from 18.3 pg.cell-1.day-1 for standard fed-batch to 39.4 pg.cell-1.day-1 for medium seeding supplemented fed-batch (2.2-fold increase) achieving a final titer of 10.8 g/L. The observations made with the two model cell lines extended to a wide panel of CHO-M-producing cell lines expressing different molecules (Fig. 4b). For the mAbs and other proteins, an average 2.1-fold increase was obtained by combining supplementation and intermediate seeding increase, while a dramatic 7.0-fold increase was obtained with medium seeding supplemented fed-batch strategy for difficult-to-express proteins (*q*_*p*_ < 2 pg.cell-1.day-1). Process intensification used in this study was, therefore, not restricted to a unique CHO-M producing cell line but rather appears to be appropriate to any CHO-M-producing cell line.

Examining the product quality attributes of mAb A and mAb B, some differences were observed when comparing the conventional fed-batch with medium seeding supplemented fed-batch (Table 1). First, mAb A and mAb B aggregation levels increased from 2.4 to 4.4% and from 1.9 to 2.7%, respectively. Such HMW levels should not be considered as problematic during process development where acceptance level is basically below 5% aggregates following a single protein A purification step [29]. No major impact was observed on product fragmentation since LMW levels remained similar for mAb A and a slight decrease from 7.3 to 5.5% was observed for mAb B. Second, some charge variants differences were observed between the two processes, following different trends for the two studied molecules (Table 1). These changes could be ascribed to analytical and process variability. Indeed, some analytical studies during manufacturing have reported a product quality acceptance range of ± 20% with respect to the abundance of charge variants [30]. Third, applying a medium seeding supplemented fed-batch process was found to moderately impact the glycan profiles. Interestingly, the Man5 level found in mAb A decreased from 7.4 to 4.0%, while remaining at similar level for mAb B. In some cases, reduction in Man5 species could be attributed to the presence of copper. Agalactosylated species remained comparable for mAb A while decreased for mAb B which was concomitant with an increase of the galactosylated form G1F from 9.6 to 14.7%. An increase in G1F glycoforms from 10.6 to 14.3% was also observed for mAb A. Increase in galactosylation was ascribed to the presence of copper and iron acting as galactosyltransferase cofactors [31]. G2F species remained at low and comparable levels for both fed-batch strategies. Based on these observations, we infer that applying a medium seeding supplemented fed-batch process cannot be considered as negatively impacting size, charge and glycan profiles (Supplementary Fig. 3).

**Table 1:** mAb A and mAb B product quality attributes comparison between conventional fed-batch and medium seeding supplemented fed-batch

Protein	Process	Size variant (%)		Charge variant (%)			N-Glycosylation (%)			
		HMW	LMW	Acidic	Main	Basic	Man5	G0	G1F	G2F
mAb A	Standard fed-batch	2.4	10.9	56.7	28.0	15.4	7.4	81.0	10.6	1.1
	Medium seeding supplemented fed-batch	4.4	11.6	50.7	35.9	13.4	4.0	80.2	14.3	1.5
mAb B	Standard fed-batch	1.9	7.3	39.0	57.4	3.6	3.5	86.1	9.6	0.8
	Medium seeding supplemented fed-batch	2.7	5.5	48.1	46.8	5.1	3.2	80.9	14.7	1.2

Analytics were performed from day 14-harvested material. G0 = proportion of agalactosylated structures (G0 = G0F + G0FN)

*HMW* High Molecular Weight, *LMW* Low Molecular Weight

**Fig. 5:**
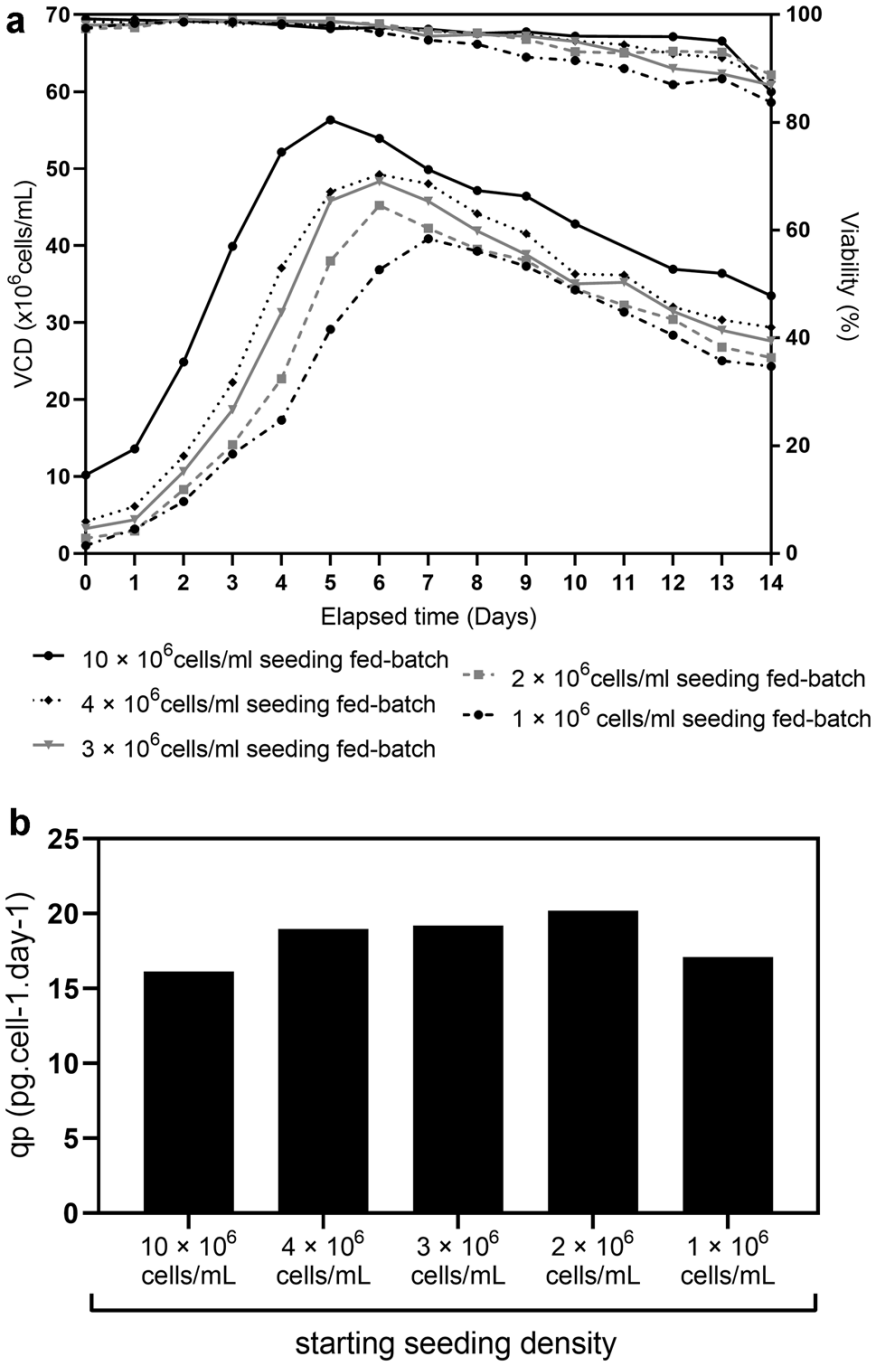
Supplemented fed-batch performance with various starting densities. VCD and viability (**a**) and specific productivity (**b**) of five fed-batches expressing mAb A starting with initial seeding density of 1, 2, 3, 4 and 10 × 10^6^ cells/mL and supplemented with copper acetate and ferric citrate

To gain greater flexibility in supplemented fed-batch culture process, we focused on developing processes working across a range of seeding densities to minimize the seeding density dependence. Figure 5a shows the cell growth and viability of fed-batch cultures expressing mAb A with adapted process parameters and feeding regimen (adapted bolus of Cell Boost 7a and Cell Boost 7b feeds in presence of supplements A and B). The fed-batches initial seeding density were 1, 2, 3, 4 and 10 × 10^6^ cells/mL, respectively. Cell density reached a peak value of 40.9 × 10^6^ cells/mL on day 7, 45.2 × 10^6^ cells/mL on day 6, 48.32 × 10^6^ cells/mL on day 6, 49.23 × 10^6^ cells/mL on day 6 and 56.31 × 10^6^ cells/mL on day 5, respectively. Similar to previous results, the VCD peak was reached 2 days post-temperature shift and occurred on day 5 for 1 × 10^6^ cells/mL seeded fed-batch, on day 4 for 2, 3, and 4 × 10^6^ cells/mL seeded fed-batch, and on day 3 for 10 × 10^6^ cells/mL seeded fed-batch. The optimized feeding conditions maintained the cells in the production state. The five fed-batches showed a similar specific productivity from day 6 to day 14 with an average *q*_*P*_ (*n *= 5) of 18.3 ± 1.6 pg.cell-1.day-1 (Fig. 5b). For all supplemented processes, key nutrients residual and by-products profiles were found to follow similar trends overtime (data not shown).

We also examined product quality attributes variation over the duration of a high-seeding supplemented fed-batch process. Interestingly, mAb A and mAb B showed low variability in terms of size, charge or glycan species between the day 7, day 10 and day 14 harvest time, as supported by the relatively low standard deviation observed (Table 2). Therefore, in such intensified process, the changes in product quality are not directly correlated with changes in cell viability or harvesting time but rather to analytical or process variability. Taken together, analytic characterization of the intensified process points out moderate and acceptable changes in product quality attributes by comparison to conventional fed-batch process and culture harvesting time.

**Table 2:** mAb A and mAb B product quality attributes comparison throughout a high-seeding supplemented fed-batch

Protein	Size variant (% ± SD)		Charge variant (% ± SD)			N-Glycosylation (% ± SD)			
	HMW	LMW	Acidic	Main	Basic	Man5	G0	G1F	G2F
mAb A	4.7 ± 0.6	9.0 ± 1.6	55.8 ± 2.0	30.8 ± 1.8	13.4 ± 0.3	5.1 ± 0.6	88.3 ± 0.7	5.1 ± 0.6	0.4 ± 0
mAb B	2.7 ± 0.1	5.5 ± 0	49.8 ± 1.6	44.1 ± 1.8	6.0 ± 0.2	5.1 ± 0	85.9 ± 0.9	2.7 ± 0.1	0.6 ± 0.1

Analytics were performed from day 7, day 10 and day 14-harvested material. Table shows the average of the three timepoints analysis where the error is the standard deviation (*n *= 3). G0 = proportion of agalactosylated structures (G0 = G0 + G0F + G0F-N), G1 = proportion of monogalactosylated structures (G1F = G1Fa + G1Fb)

*HMW* High Molecular Weight, *LMW* Low Molecular Weight

**Fig. 6:**
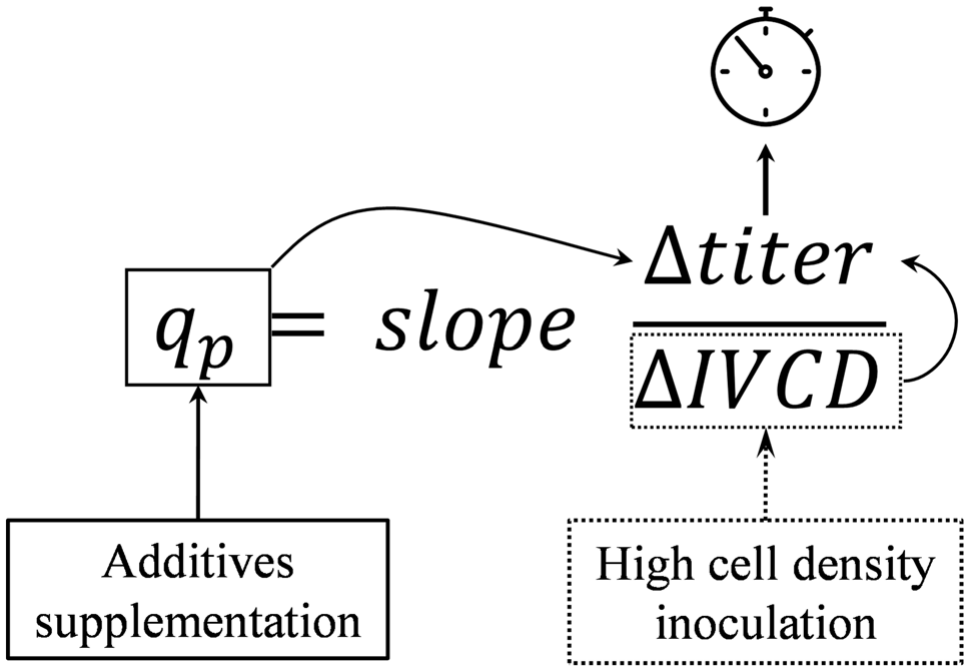
Schematic representation of key determinants for increased productivity. Specific protein production (*q*_*P*_) is a measure of protein production rate during a defined production time (from day 6 to day 14 production time in this study). *q*_*P*_ is determined from the linear slope from the plot of protein concentration versus IVCD

**Fig. 7:**
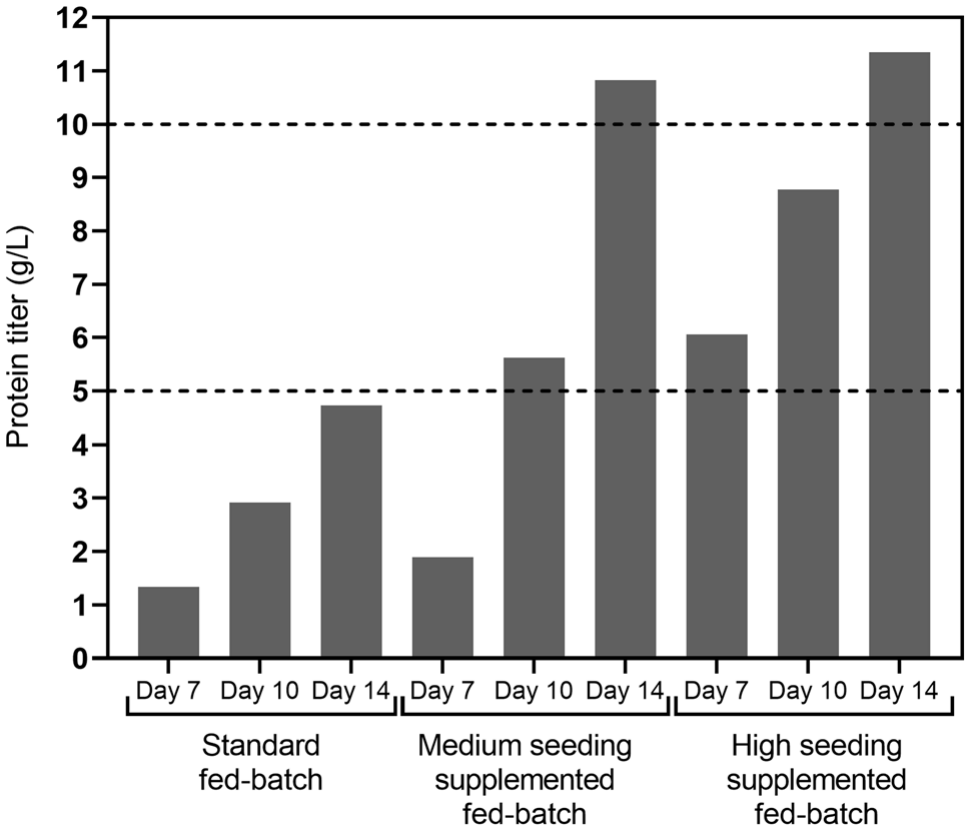
Impact of intensified fed-batch process performance on titer and time. Comparison of day 7, day 10 and day 14 harvest titer of mAb B between standard fed-batch, medium seeding density supplemented fed-batch and high seeding density supplemented fed-batch processes

Considering all the above-mentioned aspects, our results conclusively show that playing on high cell density inoculation or additive supplementation, has the potential to positively impact final harvest titer. The equation of Fig. 6 reflects our approach towards adjusting the standard fed-batch to reach the highest titers. The purpose was not only to improve productivity but also get the opportunity to adapt to the manufacturing needs. Considering a titer target of 5 g/L, production time could be reduced by 30% to 10 days for medium seeding supplemented fed-batch or by 50% to 7 days for high seeding supplemented fed-batch achieving a comparable titer as the standard fed-batch in 14 days (Fig. 7). As accelerated manufacturing production timelines can be determinant, the capacity of choosing between acceptable titer and process duration is essential. Thus, we demonstrated that process optimization developed in this study enable balancing with the equation of titer determination (Fig. 6) to better satisfy the demand of the production keeping moderate and acceptable changes in quality attributes across all the fed-batch processes regardless harvesting time.

## Discussion

Process development has become a critical stage that is studied during the development of biotherapeutics. Understanding the factors that can impact the cell culture performance and final quality attributes is equally important. In the present study, we have developed a series of fed-batch culture modes in order to provide on-demand process solutions. From supplements addition-oriented approach to intensified culture mode, we demonstrated that these processes are not CHO clone-specific and protein product-specific but rather generic solutions applicable to any producing cell line. Our different process operating conditions allowed for high cell-specific productivity, with titers above 10 g/L, while maintaining a high viable cell density over a 14-day production time. We found that increasing the initial seeding density above a threshold enabled faster proliferation rate to attain higher biomass and we successfully tightened the control of cellular needs by fine tuning this process parameter. In addition, we showed that an intelligent feeding regimen of selected additives maximized product yield without impacting product quality, whatever the duration of the selected process. Nevertheless, some product quality attributes changes were observed when comparing conventional fed-batch with medium seeding supplemented fed-batch processes. Although high molecular weight species increased up to 4.4% for mAb A, it remained at low level. This is not considered critical at early stages of process development and can be considerably reduced by subsequent purification and process steps. Other changes with respect to charge variants were highlighted and assigned to analytical and process variability. The latest being also observed during commercial development and not reported to affect the efficacy and safety of a therapeutic mAb [32].

Overall, the most important finding of this study is the concept of flexibility in the choice of fed-batch mode to adapt to the manufacturing needs. First, by maintaining a typical fed-batch operation mode, we free ourselves from being obliged to find adapted support technologies in contract manufacturing organization. Indeed, for perfusion modes or other related continuous process, specialized equipment is required and large volumes of perfusion media have to be prepared and stored [9]. Second, if space is a major problem [33], we can still shift from traditional fed-batch which may require large size bioreactors to alternative high seeding density fed-batch mode, reducing equipment footprint. In a word, a facilitated process transfer should keep things easy and should fit to any facility organization with minor changes rather than facing technical process restrictions or design [34, 35].

Subsequently, we investigated the relationship between biomass and process duration to determine the threshold between these factors that contributes the most to successful production. To do so, we carried out cultures at varying seeding density, 0.45, 1, 2, 3, 4 and 10 × 10^6^ cells/mL. Knowing the amounts of product required for the chosen indication, the stability of the final product or its intrinsic production toxicity for host cells, appropriate cell inoculation can be considered along with process duration. Clinical manufacturing batches in bioreactors of 2000 L are usually inoculated at < 0.5 × 10^6^ cells/mL with cells grown in a fed-batch mode [36]. Inoculating at 10 × 10^6^ cells/mL may require an enlarged amplification pre-stage with innovative technologies such as ATF perfusion system in the N-1 stage [13]. However, for large-scale commercial manufacturing bioreactors > 10,000 L, it could be out of consideration because not operationally achievable for the preselected manufacturing facility. The higher proliferation rate of the CHO-M cell line with maximum cell density peaking at 50 × 10^6^ cells/mL makes it now feasible to seed 10,000 L bioreactor with the N-1 fed-batch culture at day 6 considering a 10 × dilution.

Another important point to consider is timelines. In the high-stakes race to market for a novel drug release, accelerated production timelines can be determinant to circumvent competitors and turned to be more strategic than productivity and cost. Choosing an adapted short process in the early phase of cell line development avoids introducing changes afterwards which is risky and even time consuming. In this study, we demonstrated that titers can be increased by 4.5-fold at day 7 when 10 × 10^6^ cells/mL are used as inoculum with equivalent performance and quality as a 14-day run time of a conventional fed-batch. This quicker process, can generate multi-grams titer, thus saving time and reducing costs. Not only would it allow for faster delivery of the pipeline but it would also improve the facility usage management, while increasing the throughput of manufacturing batches. The process will adjust accordingly to the considerations of the drug developer. Thus, it could be adjusted to better fit the organizational or commercial interests by slightly increasing process time or decreasing seeding density, without compromising efficiency output, as shown in the study.

Biomanufacturing is evolving fast, flexibility is likely to be a key determinant in the future. By providing on-demand process solutions as soon as the cell line development path but without imposing technical restrictions, we are revising the manufacture approach by anticipating needs rather than promoting ready-to-use devoted process.

## Supplementary Information

Below is the link to the electronic supplementary material.

Supplementary file1 Supplementary Fig. 1 VCD and viability of inoculum amplification before microbioreactor inoculation. Day 0 correspond to the thawing step, four days before microbioreactor inoculation CHO-M cultures were passaged in shake flask at a seeding cell density of 0.30 × 106 cells/mL (N-1) at a volume according to process needs. (TIF 246 KB)

Supplementary file2 Supplementary Fig. 2 Feeding and sampling strategy to balance ambr15 working volume. (a) Percentage of sampling (black) and feeding (grey) of the three fed-batch processes. (b) Working volume distributions overtime of different fed-batch processes. High seeding density fed-batch (dashed grey line) and medium seeding supplemented fed-batch (dashed black line) compared to the initial standard fed-batch (black solid line). (TIF 345 KB)

Supplementary file3 Supplementary Fig. 3 mAb A and mAb B normalized changes in product quality attributes with respect to standard fed-batch process. Normalized changes are represented by the mean % of change ± standard deviation (n = 2). HMW, High Molecular Weight; LMW, Low Molecular Weight. (TIF 266 KB)

## Data Availability

The datasets generated during and/or analyzed during the current study are not publicly available due to competitive interest but are available from the corresponding author on reasonable request.

## List of symbols

IVCD _Dx-1_: Integral viable cell density at Day x-1 [10^6^ cells.days.mL^−^^1^]
q_p_: Cell-specific productivity [pg. cells^−^^1^.day^−^^1^]
t_Dx or Dx-1_: Elapsed time at Day x or Day x-1 [day]
[Titer]: Product concentration [g. L^−^^1^]
VCD_Dx or Dx-1_: Viable cell density at Day x or Day x-1 [10^6^ cells.mL^−^^1^]
