# Supplementary figures and images for: Exploring the limits of conventional small-scale CHO fed-batch for accelerated on demand monoclonal antibody production
Source: Bioprocess Biosyst Eng. 2021 Nov 9;45(2):297–307. doi: 10.1007/s00449-021-02657-w (PMC8807460; doi:10.1007/s00449-021-02657-w)

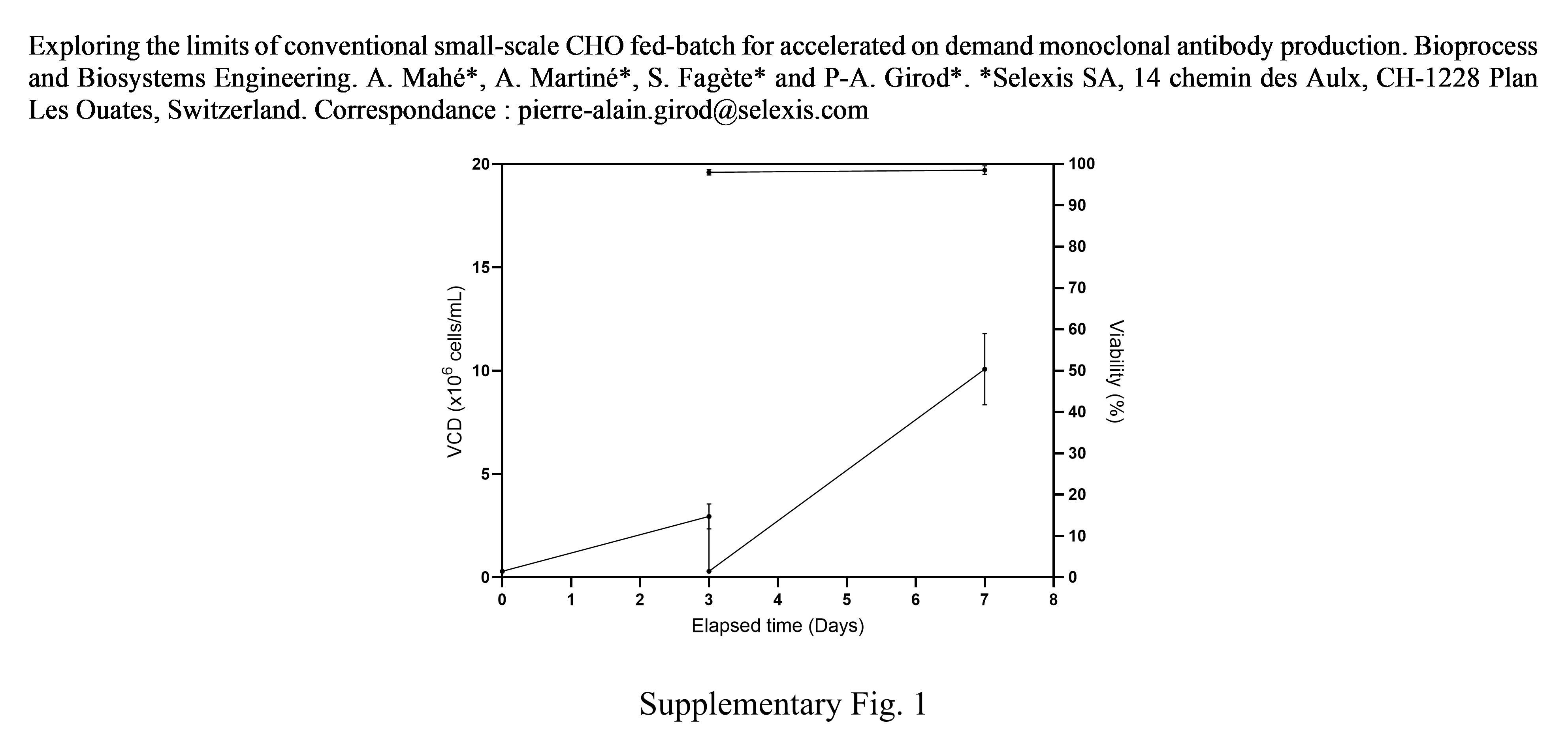

Supplement: Supplementary file 1 — Supplementary file1 Supplementary Fig. 1 VCD and viability of inoculum amplification before microbioreactor inoculation. Day 0 correspond to the thawing step, four days before microbioreactor inoculation CHO-M cultures were passaged in shake flask at a seeding cell density of 0.30 × 10^6^ cells/mL (N-1) at a volume according to process needs. (TIF 246 KB) [file 449_2021_2657_MOESM1_ESM.tif]

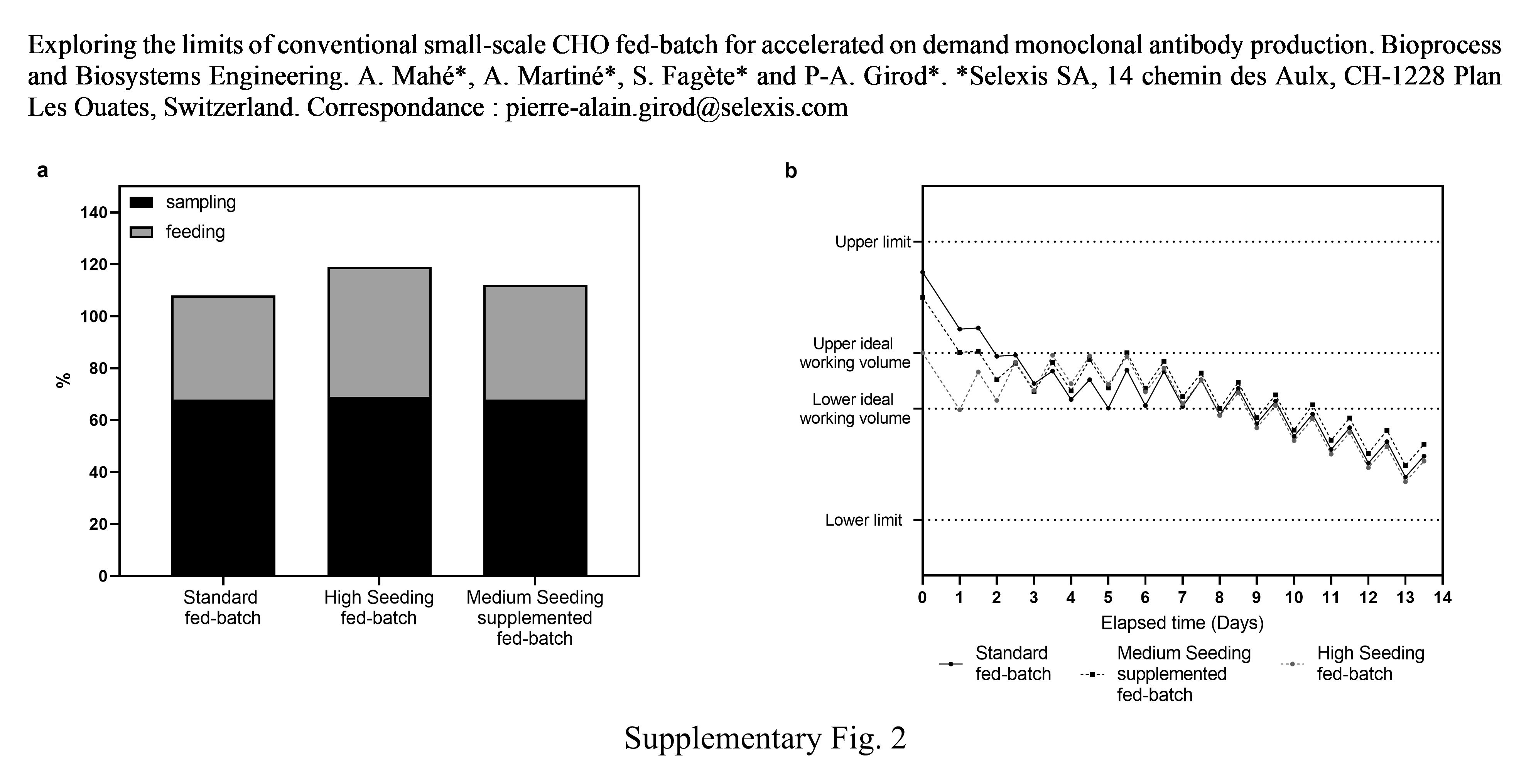

Supplement: Supplementary file 2 — Supplementary file2 Supplementary Fig. 2 Feeding and sampling strategy to balance ambr15 working volume. (a) Percentage of sampling (black) and feeding (grey) of the three fed-batch processes. (b) Working volume distributions overtime of different fed-batch processes. High seeding density fed-batch (dashed grey line) and medium seeding supplemented fed-batch (dashed black line) compared to the initial standard fed-batch (black solid line). (TIF 345 KB) [file 449_2021_2657_MOESM2_ESM.tif]

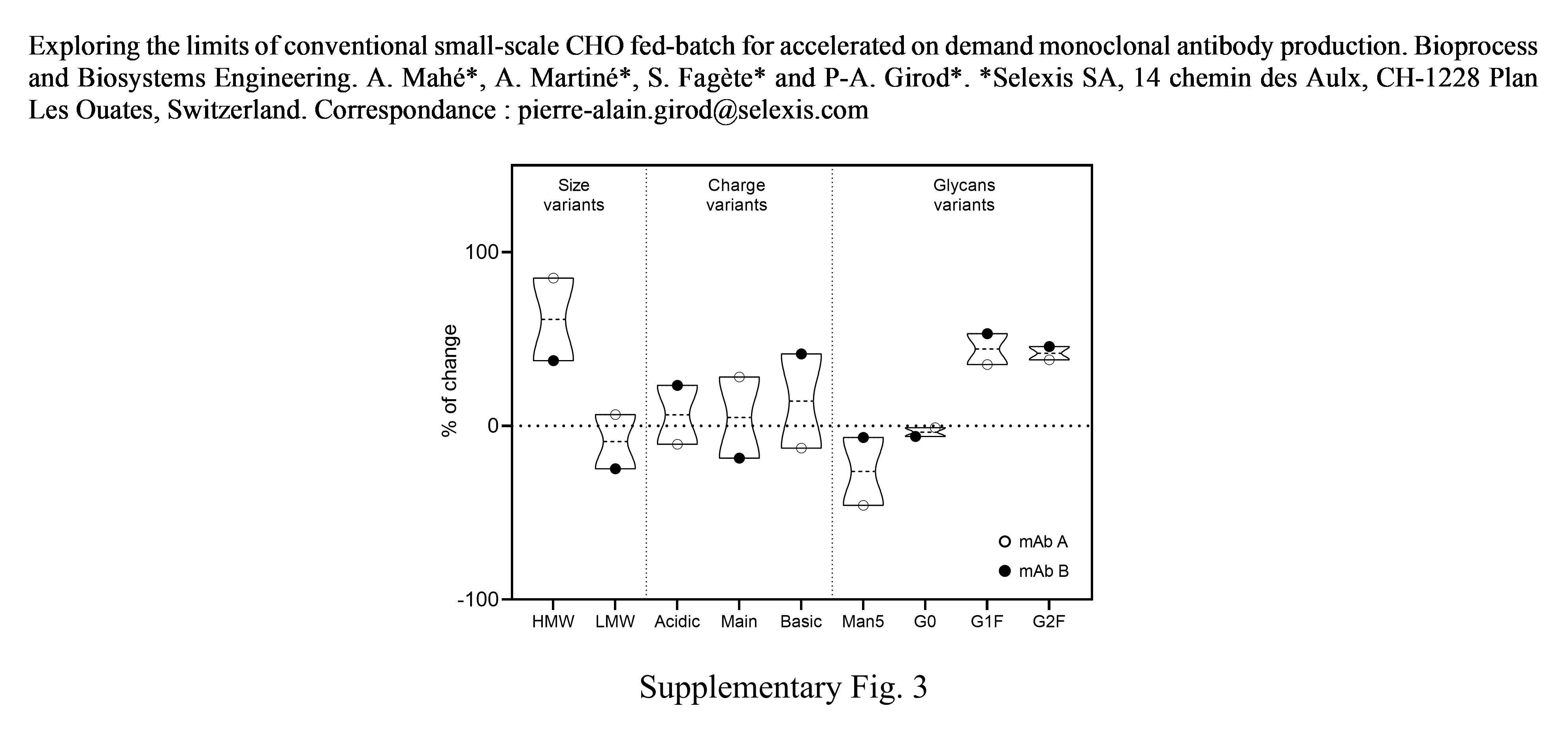

Supplement: Supplementary file 3 — Supplementary file3 Supplementary Fig. 3 mAb A and mAb B normalized changes in product quality attributes with respect to standard fed-batch process. Normalized changes are represented by the mean % of change ± standard deviation (n = 2). HMW, High Molecular Weight; LMW, Low Molecular Weight. (TIF 266 KB) [file 449_2021_2657_MOESM3_ESM.tif]
